# Serum Biomarkers of Exposure to Perfluoroalkyl Substances in Relation to Serum Testosterone and Measures of Thyroid Function among Adults and Adolescents from NHANES 2011–2012

**DOI:** 10.3390/ijerph120606098

**Published:** 2015-05-29

**Authors:** Ryan C. Lewis, Lauren E. Johns, John D. Meeker

**Affiliations:** Department of Environmental Health Sciences, University of Michigan School of Public Health, 1415 Washington Heights, Ann Arbor, MI 48109, USA; E-Mails: rclewis@umich.edu (R.C.L.); laujohns@umich.edu (L.E.J.)

**Keywords:** biomarkers, perfluoroalkyl substances, testosterone, thyroid function

## Abstract

Perfluoroalkyl substances (PFASs) are a group of environmentally-persistent chemicals that have been widely used in many industrial applications. There is human and animal evidence that PFASs may alter levels of reproductive and thyroid-related hormones. However, human studies on the potential age-related effects of PFASs on these outcomes among males and females are limited. We explored the relationship between serum PFASs and serum total testosterone (T), thyroid stimulating hormone (TSH), and free and total triiodothyronine (FT3, TT3) and thyroxine (FT4, TT4) among males and females 12 to 80 years of age from the 2011–2012 cycle of the National Health and Nutrition Examination Survey. Associations were assessed using multiple linear regression models that were stratified on sex and age categories. Effect estimates from the majority of the adjusted models were not statistically significant. However, exposure to PFASs may be associated with increases in FT3, TT3, and FT4 among adult females, but during adolescence, PFASs may be related to increases in TSH among males and decreases in TSH among females. No significant relationships were observed between PFASs and T in any of the models. These findings suggest that exposure to PFASs may disrupt thyroid hormone homeostasis.

## 1. Introduction

Perfluoroalkyl substances (PFASs) refer to a broad class of synthetic chemicals that have been widely used in a variety of industrial applications (e.g., food packaging, firefighting foams, and non-stick pan, paper, and textile coatings) due to their oil, water, and/or stain resistant properties [1,2,3]. While they provide societal benefit, there is concern, however, about the potential adverse ecological and/or human health impacts of PFASs as they are persistent in the environment, have been detected in air, surface waters, and soils and in a variety of mammals, birds, and fishes around the world, and have exhibited liver, developmental, immune, and endocrine toxicity in animal models [4,5,6,7]. The perfluorinated sulfonates and perfluorinated carboxylic acids have been the most extensively studied, namely perfluorooctane sulfonate (PFOS) and perfluorooctanoic acid (PFOA), but others like perfluorohexane sulfonic acid (PFHxS) and perfluorononanoic acid (PFNA) have been studied as well [4,5]. Sources of exposure are numerous and include industrial emissions, PFAS-containing consumer products, contaminated drinking and surface water, and house dust [1]. In 2006, the primary producers of PFASs committed to globally reducing emissions and product content of PFOA, PFOA precursors, and related higher homologue chemicals by 95% and 100% no later than 2010 and 2015, respectively [5,8]. Despite these efforts, PFASs are resistant to degradation and, consequently, exposure to them may occur long after they have been eliminated from production. However, even if the source of exposure is removed, measureable levels of PFASs may be detected in humans due to the relatively long half-life of these chemicals. The estimated half-life for PFOS, PFOA, and PFHxS in humans ranges from 3.8 to 8.5 years [5]. The half-life of PFNA has not been estimated in humans [9]. In the U.S. alone, over 95% of adolescents and adults have measureable serum levels of PFOS, PFOA, PFHxS, and PFNA [10], which may reflect current or historic exposures. In May, 2015, hundreds of scientists globally signed the Madrid Statement, which called for “limiting the production and use of PFASs and in developing safer nonfluorinated alternatives” [11].

Testosterone (T) is an important hormone for normal physiology at all life stages, and, as a result, even subtle disruptions in the levels of T could adversely impact human health. For example, T is the main sex hormone in males and is essential in the development and maintenance of secondary sexual characteristics. T also influences muscle mass, bone density, mood, and cognition [12,13]. In females, T plays a crucial role in bone metabolism and is necessary for normal ovarian, cognitive, and sexual function [14]. Joensen *et al.* [15] observed that serum concentrations of PFOS were inversely associated with total and free T, free androgen index, and reproductive hormone ratios (e.g., T/luteinizing hormone), and concentrations of PFNA were inversely related to estradiol in an analysis of young Danish men (*n* = 247, mean age (standard deviation): 19.6 (1.4) years old). No relationships were noted between levels of several other PFASs (e.g., PFOA and PFHxS) and reproductive hormones. In an earlier study of similarly aged Danish men, unadjusted median levels of serum PFOA were suggestively (*p*-value = 0.1) higher (4.4 *vs.* 5.0 ng/mL) among individuals with low testosterone compared to those with high testosterone [16]. Decreases in serum and testicular T and increases in serum estradiol have also been noted in adult rats that were dosed with PFOA [5], which, collectively with the Danish studies, suggests that PFASs may exhibit anti-androgenic effects. Nevertheless, these findings are not supported by the small number of other epidemiology studies, which reported no relationships between PFASs and T [17,18]. However, both of these epidemiology studies [17,18] did not stratify by age, and, in the Raymer *et al.* [17] study in particular, the associations between PFASs and T were assessed using unadjusted correlation coefficients, both of which may have contributed to their inability to replicate the findings of the Danish studies. Given the limited number of human studies to date, further exploration of the potential influence of PFASs on T is needed. This is especially the case for the U.S. in which no previous studies concerning PFASs and T have been conducted among males and females from the general population.

Similar to T, hormones that regulate thyroid homeostasis, including thyroid stimulating hormone (TSH), triiodothyronine (T3), and thyroxine (T4), are essential in a variety of human physiological functions including metabolism and growth and development. Toxicology studies have observed thyroid hormone imbalance in both adult and neonate rats treated with PFOS [5]. Epidemiologic investigations likewise have found that exposure to PFASs is associated with altered levels of these hormones in cohorts from around the world, including Korea [19], China [20], Canada [21,22], and the U.S. [23,24]. While the patterns are inconsistent, taken together, the animal and human evidence suggest that PFASs can alter normal thyroid function, which may have important implications for public health. The two U.S. studies used data from the 2007–2010 cycles of the National Health and Nutrition Examination Survey (NHANES), thus, there is opportunity to reexamine these relationships using the most recent NHANES data (*i.e.*, 2011–2012 cycle).

In the present study, our primary aim was to explore the relationship between serum PFASs (PFOA, PFOS, PFHxS, and PFNA) and serum T (total) among males and females 12 to 80 years old from the 2011–2012 cycle of NHANES. Our secondary motivation was to examine in this cohort the association between these select serum PFASs and serum TSH, and free and total triiodothyronine (FT3 and TT3) and thyroxine (FT4 and TT4). We decided to focus on these hormones as there is evidence from previous human and animal studies that exposure to PFASs may disrupt both the hypothalamic-pituitary-testes and hypothalamic-pituitary-thyroid axes. This study is unique because it is the first NHANES analysis of PFASs and thyroid function to stratify on age and sex, and the first NHANES analysis to explore the relationship between PFASs and T. Our approach is notable because PFASs may exhibit sex-specific effects on T and thyroid function at different times during life, which may impact the body in different ways. Given that it also relies on the most recent data, the present study may represent the effects of PFASs on T and thyroid function based on levels most representative of the current exposure burden in the U.S. population.

## 2. Methods

### 2.1. Study Population

This analysis utilized publicly-available data that was derived from NHANES 2011–2012. NHANES is a cross-sectional survey that is administered by the National Center for Health Statistics (NCHS) of the Centers for Disease Control and Prevention (CDC) that collects nationally representative data on the health and nutritional status of non-institutionalized civilian residents, and conducts more detailed laboratory analyses on a subset of the participants [25]. We analyzed data from a subset of males and females that had data on serum PFASs, T, TSH, and thyroid hormones (*n* = 1887). Data concerning measures of thyroid function were not available for participants that were <12 years old, which is why our data set was limited to participants that were 12 to 80 years old. Participants with missing data on age, sex, body mass index (BMI), race/ethnicity, poverty income ratio (PIR), and serum cotinine were excluded from the current analysis (*n* = 205), resulting in a final sample size of 1682 males and females. NHANES received approval from the NCHS Ethics Review Board, and informed consent was obtained for all participants.

### 2.2. Demographic Data

Information on family income and race was obtained from subjects in the home by a trained interviewer using a Computer-Assisted Personal Interviewing system [26]. The interviews were conducted in English or Spanish or with the assistance of an interpreter if necessary. PIR was calculated by CDC as the ratio of family income to poverty guidelines established by the Department of Health and Human Services [26].

### 2.3. Body Measurements

Height and weight were collected in a Mobile Examination Center (MEC) by trained health technicians with the assistance of a recorder during the body measurements [27]. BMI was calculated by CDC as weight in kg divided by height in m^2^ [27].

### 2.4. Laboratory Measurements

Whole venous blood samples were collected from participants at the MEC, which were then processed, stored, and shipped to laboratories at the CDC (Atlanta, GA, USA) or Collaborative Laboratory Services (Ottumwa, IA, USA) (TSH and thyroid hormones only) for analysis. Samples of serum were analyzed for the following biomarkers: cotinine, PFOA, PFOS, PFHxS, PFNA, T, FT3, TT3, FT4, TT4, and TSH. Serum cotinine/T, PFASs, and TSH/thyroid hormones were measured using isotope dilution-high performance liquid chromatography-tandem mass spectrometry (HPLC-MS/MS) [28,29], solid phase extraction-high performance liquid chromatography-turbo ion spray-tandem mass spectrometry [30], and various immunoenzymatic assays [31], respectively. While most of these variables were collected in multiple NHANES cycles, data on T measured by HPLC-MS/MS was only available for the NHANES 2011–2012 cycle, which is why we limited our analysis to those years. Additional PFASs were detected with much less frequency, so we limited our analysis to PFOA, PFOS, PFHxS, and PFNA. Concentrations below the limit of detection (LOD) were assigned a value of LOD divided by the square root of 2. LODs for the PFASs are reported in the footnotes section of Table 2 and Table 3.

### 2.5. Statistical Analysis

Statistical analysis was performed using SAS version 9.3 for Windows (SAS Institute, Cary, NC, USA). Descriptive statistics of participant demographics and concentrations of the analytes were calculated. The associations between PFASs and hormone concentrations were first assessed using simple linear regression in unadjusted models. These associations were then examined using multiple linear regression in models that were adjusted for the following variables: age, BMI, PIR, race/ethnicity, and serum cotinine. These variables were included in final models because when individually added to unadjusted models, the beta estimate for the PFAS biomarker changed by >10% and/or increased in precision for the majority of PFAS-hormone combinations. In unadjusted and adjusted models, hormones were log-transformed because they did not follow a normal distribution. Serum cotinine, age, BMI, and PIR, were modeled untransformed, whereas all PFASs were log-transformed to improve model fit. Race/ethnicity was categorized as Mexican American, other Hispanic, non-Hispanic Black, non-Hispanic White, and other/multi-racial. We chose not to use sampling weights in our analysis because when variables employed in the calculation of sampling weights are also included in statistical models, a weighted analysis can lead to decreased precision of effect estimates [32,33]. This approach has been employed in other recent studies using data from NHANES [33,34]. In NHANES 2011–2012, there was oversampling of certain racial, income, and age groups and, as a result, race, income, and age were also used in the calculation of sampling weights [35]. Because we adjusted for race, income, and age in our statistical models, an unweighted analysis was more appropriate than a weighted analysis. Regardless, by subsetting the analysis into smaller age-sex groups, adjusting for the survey methodology was not possible [36]. To facilitate interpretation of the beta coefficients, results were expressed as percent change in hormone concentration associated with a doubling (*i.e.*, 100% increase) in PFAS concentration (equation: % change = [2^beta – 1] × 100). We stratified analyses by sex and age (12 to <20, 20 to <40, 40 to <60, and 60 to 80 years old) as we hypothesized that the relationships between PFASs and hormones varied in age and/or sex-specific manners. As a sensitivity analysis, we also re-ran the models where PFAS concentrations were categorized into quartiles (the lowest quartile as the referent) to explore potential non-linear relationships. We defined statistical significance as *p* < 0.05.

## 3. Results

Table 1 shows the characteristics of the subset of participants (*n* = 1682) from NHANES analyzed in our study. Overall, this subset had a median age of 40 years, was predominantly non-Hispanic White (34%), and was equally comprised of males and females. This subset was comparable to the overall sample of participants (*n* = 1887) on age, race, and sex, as well as BMI, PIR, and serum cotinine.

**Table 1:** Characteristics of participants from NHANES 2011–2012.

Variable	Overall Sample (*n* = 1887)		Sub-Sample (*n* = 1682) ^a^	
	%	P50 (P25, P75)	%	P50 (P25, P75)
Age (years)		41 (24, 60)		40 (24, 59)
Male	51		51	
Female	49		49	
BMI (kg/m^2^)		27 (23, 32)		27 (23, 32)
Mexican American	11		11	
Other Hispanic	10		10	
Non-Hispanic White	35		36	
Non-Hispanic Black	26		26	
Other/multi-racial	18		17	
PIR (no units)		1.89 (0.99, 3.93)		1.89 (1.00, 3.93)
Serum cotinine (ng/mL)		0.04 (0.01, 1.07)		0.04 (0.01, 1.05)

P25, 25th percentile; P50, 50th percentile; P75, 75th percentile. **^a^** Individuals with data on age, sex, BMI, race/ethnicity, PIR, serum cotinine, serum PFASs, serum T, and serum thyroid hormones.

Table 2 and Table 3 show the distributions of the serum PFAS and hormone concentrations in males and females, respectively, across all age categories. PFOA, PFOS, PFHxS, and PFNA were detected in 97%–100% of the serum samples. Median concentrations of PFASs were higher in males compared with females within each age category, except for levels of PFOA among the 60 to 80 year-olds, and PFNA among the 12 to <20 and 60 to 80 year-olds, which were similar between sexes. Median concentrations of T were much higher (16–23 times higher) in males compared with females, but the levels of the other hormones were relatively comparable between sexes across all age categories.

**Table 2:** Median (25th percentile, 75th percentile) serum concentrations of PFASs and hormones among males from NHANES 2011–2012.

Analyte	Units	12 to <20 years old (*n* = 158)	20 to <40 years old (*n* = 268)	40 to <60 years old (*n* = 218)	60 to 80 years old (*n* = 213)
PFOA ^a^	ng/mL	1.85 (1.46, 2.45)	2.35 (1.87, 3.24)	2.31 (1.72, 3.39)	2.48 (1.81, 3.48)
PFOS ^b^	ng/mL	4.60 (3.12, 6.88)	7.75 (5.17, 10.80)	9.28 (5.61, 13.9)	11.1 (6.94, 18.4)
PFHxS ^c^	ng/mL	1.28 (0.77, 6.88)	1.55 (0.96, 2.53)	1.81 (1.03, 2.59)	1.72 (1.08, 2.61)
PFNA ^d^	ng/mL	0.78 (0.56, 1.19)	0.98 (0.67, 1.31)	1.00 (0.67, 1.57)	1.07 (0.77, 1.58)
T (total)	ng/dL	384.0 (240.7, 496.6)	411.9 (313.3, 507.8)	349.4 (261.1, 462.7)	345.7 (269.0, 494.3)
T3 (free)	pg/mL	3.7 (3.5, 4.0)	3.4 (3.2, 3.6)	3.3 (3.1, 3.4)	3.0 (2.7, 3.2)
T3 (total)	ng/dL	137.0 (125.0, 156.0)	119.0 (108.0, 132.0)	117.5 (105.0, 129.0)	106.0 (91.0, 122.0)
T4 (free)	ng/dL	0.8 (0.8, 0.9)	0.9 (0.8, 0.9)	0.8 (0.7, 0.9)	0.9 (0.8, 0.9)
T4 (total)	µg/mL	7.5 (6.8, 8.3)	7.7 (6.7, 8.5)	7.8 (7.0, 8.8)	8.0 (7.0, 9.1)
TSH	µIU/mL	1.4 (1.1, 2.1)	1.4 (1.0, 1.9)	1.4 (1.1, 2.1)	1.7 (1.2, 2.5)

^a^ 99%–100% ≥ LOD (0.10 ng/mL); ^b^ 99%–100% ≥ LOD (0.20 ng/mL); ^c^ 98%–100% ≥ LOD (0.10 ng/mL); ^d^ 99%–100% ≥ LOD (0.08 ng·mL).

**Table 3:** Median (25th percentile, 75th percentile) serum concentrations of PFASs and hormones among females from NHANES 2011–2012.

Analyte	Units	12 to <20 years old (*n* = 145)	20 to <40 years old (*n* = 257)	40 to <60 years old (*n* = 224)	60 to 80 years old (*n* = 199)
PFOA ^a^	ng/mL	1.53 (1.15, 2.15)	1.49 (0.95, 2.25)	1.62 (1.21, 2.39)	2.55 (1.78, 3.44)
PFOS ^b^	ng/mL	3.76 (2.33, 5.57)	4.20 (2.68, 6.62)	4.93 (2.75, 8.18)	9.50 (5.51, 14.50)
PFHxS ^c^	ng/mL	0.83 (0.56, 1.74)	0.69 (0.43, 1.10)	0.87 (0.52, 1.33)	1.47 (0.9, 2.34)
PFNA ^d^	ng/mL	0.73 (0.52, 1.10)	0.72 (0.53, 1.02)	0.78 (0.56, 1.20)	1.06 (0.77, 1.72)
T (total)	ng/dL	23.3 (16.4, 31.4)	26.0 (18.3, 37.9)	16.8 (12.1, 24.8)	15.0 (9.7, 23.0)
T3 (free)	pg/mL	3.4 (3.2, 3.7)	3.2 (3.0, 3.4)	3.0 (2.8, 3.2)	2.9 (2.8, 3.1)
T3 (total)	ng/dL	125.0 (113.0, 142.0)	117.0 (106.0, 134.0)	109.5 (96.0, 120.0)	106.0 (95.0, 119.0)
T4 (free)	ng/dL	0.8 (0.8, 0.9)	0.8 (0.8, 0.9)	0.8 (0.7, 0.9)	0.9 (0.8, 0.9)
T4 (total)	µg/mL	7.7 (7.1, 8.9)	8.0 (7.2, 9.0)	8.0 (7.2, 9.0)	8.3 (7.5, 9.4)
TSH	µIU/mL	1.4 (0.9, 1.9)	1.5 (1.0, 2.1)	1.5 (1.1, 2.1)	1.7 (1.2, 2.5)

^a^ 99%–100% ≥ LOD (0.10 ng/mL); ^b^ 99%–100% ≥ LOD (0.20 ng/mL); ^c^ 97%–99% ≥ LOD (0.10 ng/mL); ^d^ 98%–100% ≥ LOD (0.08 ng·mL).

Table 4 and Table 5 present the adjusted percent change in serum hormone concentrations associated with a doubling (100% increase) in serum PFAS concentration among males and females, respectively, across all age categories. Effect estimates from the unadjusted models are available in Supplementary Materials Tables S1 and S2. For the adjusted models, no statistically significant associations were observed between PFASs and T for males and females, and the majority of effect estimates for thyroid hormones were also not statistically significant. However, among 12 to <20 year-olds, PFOS (12.3%, 95% CI: 0.7, 25.2%) and PFNA (16.3%, 95% CI: 4.0, 30.2%) were positively associated with TSH in males, whereas PFOA (−16.6%, 95% CI: −28.6, −2.6%) was inversely related with TSH in females. PFOA (2.0%, 95% CI: 0.0, 4.1%), PFOS (2.2%, 95% CI: 0.5, 3.9%), and PFNA (2.4%, 95% CI: 0.4, 4.4%) were also positively related to FT4 among 20 to <40 year-old females, and PFOA was also positively related to FT3 (1.8, 95% CI: 0.2, 3.4) and TT3 (3.3, 95% CI: 0.6, 6.0%) among 60 to 80 year-old females. When PFAS levels were categorized into quartiles, the results corroborated the findings of the continuous measures analysis (data not shown).

**Table 4:** Percent change (95% CI) in serum hormone concentration associated with a doubling (100% increase) in serum PFAS concentration among males from NHANES 2011–2012 (adjusted results).

Hormone	PFAS	12 to <20 years old (*n* = 158) ^a^	20 to <40 years old (*n* = 268) ^a^	40 to <60 years old (*n* = 218) ^a^	60 to 80 years old (*n* = 213) ^a^
T (total)	PFOA	17.3 (−9.4, 51.8)	−0.5 (−5.0, 4.1)	−1.4 (−7.9, 5.5)	7.2 (−1.9, 17.1)
	PFOS	7.9 (−9.1, 28.1)	−1.2 (−5.1, 2.9)	−2.7 (−7.3, 2.2)	4.9 (−1.9, 12.1)
	PFHxS	2.4 (−9.1, 15.2)	−1.2 (−4.7, 2.4)	−3.6 (−8.2, 1.2)	3.3 (−3.8, 10.8)
	PFNA	1.8 (−14.9, 21.7)	0.9 (−4.1, 6.1)	−0.6 (−6.2, 5.3)	5.6 (−3.9, 16.0)
T3 (free)	PFOA	0.8 (−2.2, 3.9)	−0.2 (−1.4, 0.9)	−0.2 (−1.8, 1.4)	0.1 (−1.4, 1.6)
	PFOS	−0.3 (−2.3, 1.7)	0.0 (−1.0, 1.0)	0.7 (−0.4, 1.9)	−0.5 (−1.5, 0.6)
	PFHxS	0.0 (−1.4, 1.4)	0.1 (−0.8, 1.0)	−0.6 (−1.7, 0.6)	−0.7 (−1.8, 0.5)
	PFNA	0.3 (−1.8, 2.4)	0.2 (−1.0, 1.5)	1.1 (−0.3, 2.4)	−0.3 (−1.8, 1.3)
T3 (total)	PFOA	−1.9 (−6.4, 2.9)	−0.8 (−2.7, 1.2)	0.5 (−2.3, 3.5)	1.4 (−1.4, 4.2)
	PFOS	−1.6 (−4.7, 1.5)	−0.0 (−1.8, 1.8)	0.2 (−1.8, 2.3)	−0.6 (−2.7, 1.4)
	PFHxS	0.2 (−1.9, 2.4)	0.5 (−1.1, 2.0)	1.0 (−1.1, 3.1)	−1.3 (−3.4, 0.9)
	PFNA	−1.1 (−4.2, 2.2)	−0.0 (−2.2, 2.2)	0.5 (−1.9, 3.0)	0.3 (−2.6, 3.2)
T4 (free)	PFOA	−0.6 (−4.9, 4.0)	−0.2 (−2.0, 1.8)	−1.9 (−4.7, 0.9)	0.4 (−1.9, 2.8)
	PFOS	0.6 (−2.4, 3.6)	−0.5 (−2.1, 1.2)	0.2 (−1.9, 2.3)	0.8 (−1.0, 2.6)
	PFHxS	−1.5 (−3.5, 0.5)	−0.7 (−2.2, 0.8)	0.4 (−1.7, 2.5)	0.5 (−1.4, 2.4)
	PFNA	2.6 (−0.5, 5.8)	0.6 (−1.5, 2.8)	−1.1 (−3.5, 1.3)	0.5 (−2.0, 3.1)
T4 (total)	PFOA	−1.6 (−6.6, 3.6)	−1.2 (−3.4, 1.1)	−3.1 (−6.2, 0.1) *	−0.5 (−3.4, 2.4)
	PFOS	−1.1 (−4.4, 2.3)	−1.5 (−3.4, 0.5)	−0.8 (−3.1, 1.5)	−0.6 (−2.8, 1.6)
	PFHxS	−2.1 (−4.4, 0.2) *	−0.3 (−2.1, 1.5)	−0.7 (−3.0, 1.7)	−1.3 (−3.5, 1.0)
	PFNA	0.6 (−2.9, 4.3)	−0.5 (−2.9, 2.1)	−2.5 (−5.2, 0.2) *	−0.4 (−3.4, 2.7)
TSH	PFOA	9.6 (−7.1, 29.4)	0.1 (−6.5, 7.2)	0.2 (−10.4, 12.1)	−0.7 (−10.1, 9.7)
	PFOS	12.3 (0.7, 25.2) **	−2.9 (−8.6, 3.2)	−1.3 (−8.9, 7.1)	−2.3 (−9.4, 5.3)
	PFHxS	6.2 (−1.5, 14.5)	−0.4 (−5.6, 5.1)	0.6 (−7.2, 9.0)	−3.6 (−10.9, 4.4)
	PFNA	16.3 (4.0, 30.2) **	−0.5 (−7.7, 7.3)	0.9 (−8.3, 11.0)	0.4 (−9.7, 11.7)

**^a^** Adjusted for age (continuous), BMI (continuous), PIR (continuous), serum cotinine (continuous), and race/ethnicity (categorical). * 0.05 ≤ *p* < 0.10. ** *p* < 0.05.

**Table 5:** Percent change (95% CI) in serum hormone concentration associated with a doubling (100% increase) in serum PFAS concentration among females from NHANES 2011–2012 (adjusted results).

Hormone	PFAS	12 to <20 years old (*n* = 145) ^a^	20 to <40 years old (*n* = 257) ^a^	40 to <60 years old (*n* = 224) ^a^	60 to 80 years old (*n* = 199) ^a^
T (total)	PFOA	−10.6 (−21.5, 1.8) *	3.4 (−4.3, 11.8)	−5.4 (−13.4, 3.3)	1.8 (−8.2, 12.8)
	PFOS	−6.9 (−14.7, 1.6)	2.0 (−4.4, 8.7)	−0.4 (−6.7, 6.4)	6.7 (−1.2, 15.4)
	PFHxS	−5.3 (−11.6, 1.5)	−3.3 (−8.7, 2.5)	−2.4 (−8.7, 4.3)	−0.2 (−8.3, 8.7)
	PFNA	−9.6 (−18.7, 0.5) *	6.3 (−1.5, 14.8)	−1.9 (−10.4, 7.4)	3.5 (−5.5, 13.5)
T3 (free)	PFOA	1.1 (−2.4, 4.7)	0.4 (−1.1, 1.9)	−0.0 (−1.6, 1.5)	1.8 (0.2, 3.4) **
	PFOS	−1.1 (−3.3, 1.3)	0.5 (−0.7, 1.8)	−0.4 (−1.5, 0.8)	1.1 (−0.1, 2.2) *
	PFHxS	−0.8 (−2.6, 1.1)	0.5 (−0.6, 1.7)	−0.0 (−1.2, 1.1)	1.0 (−0.2, 2.3)
	PFNA	−1.9 (−4.7, 0.9)	0.6 (−0.9, 2.1)	0.8 (−0.8, 2.4)	1.0 (−0.4, 2.4)
T3 (total)	PFOA	0.3 (−4.2, 5.1)	−0.3 (−2.8, 2.4)	−0.5 (−3.3, 2.5)	3.3 (0.6, 6.0) **
	PFOS	−2.3 (−5.2, 0.8)	0.2 (−1.9, 2.4)	−1.3 (−3.4, 0.9)	1.1 (−1.0, 3.1)
	PFHxS	−1.3 (−3.7, 1.1)	1.0 (−1.0, 3.0)	0.8 (−1.4, 3.0)	2.0 (−0.2, 4.3) *
	PFNA	−1.6 (−5.3, 2.2)	−0.8 (−3.4, 1.8)	−1.4 (−4.3, 1.6)	1.9 (−0.5, 4.3)
T4 (free)	PFOA	2.1 (−2.2, 6.7)	2.0 (0.0, 4.1) **	1.2 (−1.3, 3.8)	−2.0 (−4.6, 0.7)
	PFOS	0.1 (−2.8, 3.1)	2.2 (0.5, 3.9) **	1.3 (−0.5, 3.2)	−0.5 (−2.5, 1.5)
	PFHxS	0.2 (−2.1, 2.6)	1.3 (−0.2, 2.8) *	0.5 (−1.4, 2.4)	−2.0 (−4.2, 0.1) *
	PFNA	−2.6 (−6.0, 0.9)	2.4 (0.4, 4.4) **	2.1 (−0.5, 4.8)	−1.5 (−3.8, 0.9)
T4 (total)	PFOA	4.1 (−0.6, 8.9) *	−0.1 (−2.7, 2.6)	0.8 (−2.2, 3.9)	−0.9 (−3.4, 1.6)
	PFOS	−0.3 (−3.4, 2.8)	−0.0 (−2.1, 2.2)	−0.7 (−2.9, 1.6)	0.2 (−1.7, 2.2)
	PFHxS	−0.3 (−2.8, 2.1)	−0.6 (−2.5, 1.4)	0.9 (−1.4, 3.2)	−0.7 (−2.8, 1.4)
	PFNA	−3.2 (−6.7, 0.5) *	0.0 (−2.5, 2.6)	−0.2 (−3.2, 3.0)	0.0 (-2.2, 2.4)
TSH	PFOA	−16.6 (−28.6, −2.6) **	0.7 (−7.7, 10.0)	3.5 (−6.3, 14.4)	4.6 (−6.6, 17.0)
	PFOS	−6.6 (−16.0, 3.8)	−1.0 (−7.9, 6.4)	0.0 (−7.1, 7.7)	−1.5 (−9.6, 7.3)
	PFHxS	−4.1 (−11.8, 4.3)	−0.1 (−6.4, 6.7)	5.0 (−2.5, 13.2)	−0.3 (−9.1, 9.3)
	PFNA	4.2 (−8.4, 18.5)	−2.5 (−10.6, 6.4)	1.7 (−8.2, 12.7)	2.8 (−7.0, 13.7)

**^a^** Adjusted for age (continuous), BMI (continuous), PIR (continuous), serum cotinine (continuous), and race/ethnicity (categorical). * 0.05 ≤ *p* < 0.10. ** *p* < 0.05.

## 4. Discussion

In this study, we assessed the associations between serum biomarkers of exposure to select PFASs (PFOA, PFOS, PFHxS, and PFNA) and serum T, TSH, and thyroid hormones (FT3, TT3, FT4, and TT4) in male and female adults and adolescents from NHANES 2011–2012. Statistically significant relationships were observed for PFASs and FT3, TT3, and FT4 among adult females and for PFASs and TSH in a sex-specific manner among adolescents. Despite these findings suggesting that exposure to PFASs may disturb normal thyroid function, the effect estimates from the majority of our models were not statistically significant. Consequently, caution should be used when interpreting our findings.

Comparing NHANES 1999–2000 (*n* = 1562) with NHANES 2003–2004 (*n* = 2094), there were statistically significant reductions in geometric mean serum levels of PFOA, PFOS, and PFHxS and statistically significant increases in geometric mean serum levels of PFNA among the U.S. population [2]. Based on comparison with NHANES 2007–2010 (*n* = 1181) [24], our results that relied on data from NHANES 2011–2012 suggest that geometric mean serum concentrations of PFOA (4.15 *vs.* 1.98 ng/mL), PFOS (14.2 *vs.* 6.21), PFHxS (2.00 *vs.* 1.19 ng/mL), and PFNA (1.54 *vs.* 0.91 ng/mL) have all decreased in Americans. Given this decreasing trend in serum levels of PFASs among the U.S. population, it may be informative to compare our results with those of earlier NHANES cycles using the same sex-age strata to see if exposure level modifies the observed associations. Aside from sex and age, other studies have also demonstrated that race, education, and pregnancy-related factors (e.g., parity and breastfeeding duration) may modify PFAS exposure [24,37] and, as a result, should be explored as effect modifiers in future studies as well.

Outside of the U.S., several studies have reported on concentrations of serum PFASs in males and females. For example, in 12–19 year-old Koreans (*n* = 77, sample year: 2008), median levels of PFOA, PFOS, PFHxS, and PFNA were 1.94, 4.57, 0.85, and 1.40 ng/mL, respectively [19]. In this same age strata, median levels for males and females collectively from our analysis were lower for PFOA (1.71 ng/mL), PFOS (4.19 ng/mL), and PFNA (0.75 ng/mL), and higher for PFHxS (1.05 ng/mL). In 17–37 year-old pregnant Japanese women (*n* = 15, sample year: 2003) [38], median concentrations of PFOS were higher compared with women in the same age range from our analysis (8.1 *vs.* 5.57 ng/mL). In addition, in young Danish men (*n* = 247, sample years: 2008–2009) [15], median levels of PFOA (3.02 *vs.* 2.25 ng/mL), PFOS (7.79 *vs.* 6.42 ng/mL), and PFNA (1.07 *vs.* 0.95 ng/mL) were higher, whereas PFHxS (0.67 *vs.* 1.23 ng/mL) was lower relative to similarly aged men from our analysis. These differences by country may reflect geographic variability in the types of sources that intentionally contain or are contaminated with PFASs. This, combined with variability in the lifestyle behaviors in different age or sex groups, may lead to different interactions with PFAS sources and, consequently, variations in the intensity, frequency, and duration of exposures.

Consistent with our findings, several other human studies have reported no associations between PFASs and T. These epidemiologic investigations were examined in adult males from Greenland/Poland/Ukraine (*n* = 197–208) [18] and the U.S. (*n* = 256) [17]. However, other studies among adult males have reported inverse associations between serum levels of PFASs and T [15,16]. Several animal studies have corroborated these findings showing decreased serum and testicular T levels following administration of PFASs to male rats [39,40,41]. Given the current conflicting epidemiological evidence, perhaps due to analytical limitations present in previous human studies (e.g., lack of age stratification in analyses or presentation of unadjusted correlation coefficients only), further epidemiological investigations are warranted not only in men, but also in women and adolescents.

Puberty induces profound changes in thyroid hormone levels to compensate for physiological demands associated with growth and sexual development [42]. Among adolescents, we observed that the relationship between PFASs and TSH levels varied by sex. No associations were observed between PFASs and FT3, TT3, FT4, and TT4 levels. It is possible that the differences observed in the effect of PFAS exposure on TSH levels between adolescent girls and boys may be due in part to the dramatic increases in sex hormones during puberty. Additional prospective studies are necessary to characterize potential PFAS-induced changes in circulating thyroid hormone levels among pubertal boys and girls. While overt thyroid disease during adolescence has a direct effect on various aspects of puberty [43,44,45,46], less is known about the hormone-mediated health effects of subclinical alterations in thyroid hormone levels during puberty. It is possible that the even subtle PFAS-induced disturbances in these hormones during this stage of development may have profound effects on growth and reproduction.

On the other hand, among adult women, we observed positive associations between PFASs and FT3, TT3, and FT4. No associations were observed in adult men. Subclinical thyroid disease in adulthood may have a significant impact on morbidity as it has been associated with an array of chronic diseases (e.g., cardiovascular disease and insulin resistance) and psychiatric disorders (e.g., depression) [47,48,49,50]. In addition, our results indicate that PFAS-associated thyroid hormone alterations in females may vary by age of exposure, which has implications for studies aimed at identifying periods of susceptibility to thyroidal disturbances across the life course. The mechanisms through which PFASs may act to influence thyroid hormone levels in both adolescents and adults are potentially varied. Animal and cellular studies have shown that PFASs may disturb thyroid hormone homeostasis along multiple points of the hypothalamic-pituitary-thyroid axis [51]. As such, PFASs may alter thyroid hormone synthesis, transport, metabolism, and/or action on target cells, all of which are involved with the regulation of thyroid hormone homeostasis [52].

The results of the human studies to date have been somewhat inconsistent with respect to the direction of the observed associations between PFASs and TSH and thyroid hormone levels. Two other studies have explored the potential relationship between PFASs and thyroid function using data from NHANES. Wen *et al.* [24] analyzed data from adult men (*n* = 672) and women (*n* = 509) in NHANES cycles 2007–2010. They reported that PFOA was positively associated with TT3 in females, and PFHxS was positively related to TT3 and TT4 in females and inversely related to FT4 in males. In the second study, Jain [23] found that among participants >12 years old from NHANES 2007–2008, PFOA was positively associated with TSH and TT3, and PFHxS was positively associated with TT4. Contrary to our study, the NHANES analyses by Wen *et al.* [24] and Jain [23] did not stratify by age, which our analysis suggests may be an important factor to consider in the toxicity of PFASs on thyroid function. Furthermore, in cohorts of Chinese adolescents and young adults (*n* = 545) [20] and pregnant Canadian women (*n* = 151) [22], positive relationships between PFNA and FT4 and TSH, respectively, were noted. Dallaire *et al.* [21] also reported that PFOA was positively associated with FT4 and inversely related to TT3 and TSH in adult Inuit (*n* = 621). Although not measured in NHANES, perfluorotridecanoic acid has been linked to decreased TT4 and increased TSH in Korean females (*n* = 633) [19]. While most of the human studies to date have focused on adults and adolescents, a few have observed altered levels of TSH and thyroid hormones in newborns from the Netherlands [53] and Korea [54] in relation to prenatal exposure to PFASs.

Contrary to these human studies, Inoue *et al.* [38] and Bloom *et al.* [55] observed no relationship between PFASs and thyroid function using data collected in Japanese infants (*n* = 15) and American adults (*n* = 31), respectively. However, the robustness and generalizability of these studies is limited given the small sample size of each cohort. Aside from changes to hormone levels, several studies have also explored the effects of PFASs on thyroid-related conditions. Specifically, no associations were observed in a cohort of pregnant Canadian women (*n* = 271) between PFOA, PFOS, or PFHxS and hypothyroxinemia [56], but a positive relationship was reported between PFOA and PFOS and current thyroid disease in adults from the U.S. using data from NHANES cycles 1999–2000 and 2003–2006 [52].

Strengths of our study included analysis of serum T by HPLC-MS/MS, which provided greater sensitivity than commonly-used immunoassay-based methods [57] used in earlier NHANES cycles, a large sample size, and the first of its kind to explore the potential relationship between PFASs and T and stratify by age categories using the most recently available data from NHANES (which helped to identify sex-specific associations of PFASs with TSH among adolescents). Limitations included the cross-sectional study design, which limited conclusions of causality due to temporal ambiguity, the large number of comparisons made, which may have led to some significant findings due to chance alone, and the lack of data on free T and sex-hormone binding globulin, which precluded further statistical analyses. In addition, it is possible that our findings among the adults may be related to the age categories selected. As a sensitivity analysis, we reanalyzed the adult data using 20 to <50 and 50 to 80 years old as the age cutoffs instead of 20 to <40, 40 to <60, and 60 to 80 years old and the results that we found in younger and older adults were similar in direction and magnitude as the original analysis (see Supplementary Materials Tables S3 and S4), which lends credibility to our original age strata. Because we chose not to use sampling weights in our analysis, the results may not be fully generalizable to males and females of the same age range from the general U.S. population. While others [24] adjusted their statistical models for iodine, we do not believe that our decision to not adjust for this variable confounded the observed relationships. In the age-sex strata in which we observed significant associations, Spearman correlations between relevant PFASs and creatinine-corrected urinary iodine were weak [median (range) of the absolute values: 0.05 (0.01–0.18)]. Thus, the possibility of residual confounding due to urinary iodine is limited. Furthermore, Spearman correlations between pairs of PFASs were moderately strong and positive [median (range): 0.62 (0.40–0.63)]. Therefore, confounding due to co-exposures to other PFASs, as well as other common environmental chemicals (e.g., polychlorinated biphenyls, phthalates, and flame retardants) that have been linked to altered thyroid function [58], cannot be ruled out. In addition, while we did not include time of blood sample collection as a covariate in our relevant models, both T [59] and TSH [60] have circadian variation in humans. However, because this covariate would only be associated with the outcome of interest and not the exposure, our approach should only reduce the precision of the effect estimates in our relevant models and not introduce any confounding bias. Lastly, it is possible that the associations observed in this study may be influenced by the interference of PFOS with serum binding proteins when using an analog method for FT4 determination, which has been shown to result in artificially decreased serum FT4 in rats highly exposed to PFOS [61]. However, the findings of this animal study were not corroborated by a subsequent study conducted in a sample of U.S. adults [62]. Thus, any interference by PFOS on FT4 in our analysis is expected to be minimal.

## 5. Conclusions

The results from this study suggest that exposure to PFASs are associated with increased thyroid hormone levels in adult females. However, exposure to PFASs may exhibit sex-specific effects on TSH during adolescence. Future studies should consider the potential age- and sex-specific effects of PFASs on thyroid function as this will help to tease apart the identified relationships. 

## Supplementary Files

Supplementary File 1
