# Supplementary material for: Serum Biomarkers of Exposure to Perfluoroalkyl Substances in Relation to Serum Testosterone and Measures of Thyroid Function among Adults and Adolescents from NHANES 2011–2012
Source: Int J Environ Res Public Health. 2015 May 29;12(6):6098–114. doi: 10.3390/ijerph120606098 (PMC4483690; doi:10.3390/ijerph120606098)
Supplement: Supplementary File 1 [file ijerph-12-06098-s001.pdf]

Serum Biomarkers of Exposure to Perfluoroalkyl Substances in Relation to Serum Testosterone and Measures of Thyroid Function among Adults and Adolescents from NHANES 2011–2012

Table S1. Percent change (95% CI) in serum hormone concentration associated with a doubling (100% increase) in serum PFAS concentration among males from NHANES 2011–2012 (unadjusted results).

Hormone	PFAS	12 to <20 years old (*n* = 158) [a]	20 to <40 years old (*n* = 268) [a]	40 to <60 years old (*n* = 218) [a]	60 to 80 years old (*n* = 213) [a]
T (total)	PFOA	33.4 (1.6, 75.2) **	−0.1 (−4.9, 5.1)	−1.5 (−8.1, 5.5)	7.2 (−1.8, 17.2)
	PFOS	19.6 (−0.2, 43.3) *	−1.0 (−5.2, 3.4)	−1.1 (−6.0, 4.0)	4.8 (−1.9, 12.0)
	PFHxS	1.4 (−10.9, 15.4)	−0.9 (−4.5, 2.9)	−3.8 (−8.6, 1.2)	3.1 (−3.9, 10.5)
	PFNA	10.4 (−8.9, 33.7)	1.1 (−4.5, 6.9)	1.6 (−4.0, 7.5)	8.7 (−0.8, 19.1)*
T3 (free)	PFOA	−0.9 (−4.1, 2.4)	−0.5 (−1.7, 0.6)	0.0 (−1.5, 1.6)	−0.1 (−1.6, 1.4)
	PFOS	−2.1 (−4.3, −0.0) *	−0.5 (−1.5, 0.5)	0.5 (−0.7, 1.6)	−0.8 (−1.9, 0.3)
	PFHxS	−0.2 (−1.7, 1.4)	−0.2 (−1.1, 0.6)	−0.5 (−1.6, 0.7)	−1.0 (−2.2, 0.1)*
	PFNA	−1.2 (−3.5, 1.1)	−0.1 (−1.4, 1.2)	0.8 (−0.5, 2.1)	0.3 (−1.3, 1.9)
T3 (total)	PFOA	−5.3 (−10.1, −0.2) **	−1.3 (−3.2, 0.7)	0.6 (−2.2, 3.4)	1.0 (−1.8, 3.9)
	PFOS	−4.8 (−8.0, −1.5) **	−0.8 (−2.5, 0.9)	0.2 (−1.9, 2.3)	−0.9 (−3.0, 1.2)
	PFHxS	−0.3 (−2.7, 2.2)	−0.0 (−1.5, 1.5)	1.1 (−1.0, 3.2)	−1.8 (−4.0, 0.4)
	PFNA	−3.5 (−6.9, 0.1) *	−0.4 (−2.7, 1.9)	0.4 (−1.8, 2.8)	1.2 (−1.7, 4.2)
T4 (free)	PFOA	−0.8 (−4.9, 3.3)	−0.1 (−2.0, 1.8)	−2.7 (−5.3, 0.1) *	0.5 (−1.7, 2.8)
	PFOS	0.8 (−1.9, 3.6)	−0.8 (−2.4, 0.8)	0.4 (−1.7, 2.5)	0.8 (−0.9, 2.5)
	PFHxS	−1.4 (−3.3, 0.5)	−1.1 (−2.5, 0.3)	−0.1 (−2.1, 2.0)	0.5 (−1.3, 2.3)
	PFNA	2.7 (−0.2, 5.6) *	0.6 (−1.5, 2.8)	−0.1 (−2.4, 2.3)	0.8 (−1.5, 3.2)
T4 (total)	PFOA	−3.3 (−7.8, 1.6)	−1.5 (−3.6, 0.8)	−3.5 (−6.5, −0.4) **	−1.1 (−3.8, 1.8)
	PFOS	−1.4 (−4.6, 1.8)	−1.9 (−3.8, −0.0) *	−0.8 (−3.2, 1.6)	−1.1 (−3.2, 1.0)
	PFHxS	−2.1 (−4.3, 0.1) *	−0.7 (−2.4, 1.0)	−0.9 (−3.2, 1.5)	−1.9 (−4.0, 0.3) *
	PFNA	0.6 (−2.7, 4.1)	−0.6 (−3.0, 2.0)	−1.9 (−4.5, 0.7)	−0.6 (−3.5, 2.4)
TSH	PFOA	8.4 (−7.7, 27.4)	0.7 (−5.9, 7.7)	0.3 (−9.7, 11.4)	−2.1 (−11.3, 8.0)
	PFOS	15.1 (3.7, 27.8) **	−3.1 (−8.6, 2.7)	−2.0 (−9.4, 5.9)	−2.8 (−9.7, 4.7)
	PFHxS	8.7 (0.9, 17.1) **	−0.1 (−5.1, 5.1)	0.2 (−7.4, 8.4)	−3.9 (−11.0, 3.9)
	PFNA	16.0 (4.0, 29.5) **	−1.0 (−8.3, 6.8)	0.5 (−7.9, 9.6)	−4.7 (−14.0, 5.5)

* $0.05 \leq p < 0.10$. ** $p < 0.05$.

Table S2. Percent change (95% CI) in serum hormone concentration associated with a doubling (100% increase) in serum PFAS concentration among females from NHANES 2011–2012 (unadjusted results).

Hormone	PFAS	12 to <20 years old (*n* = 145) [a]	20 to <40 years old (*n* = 257) [a]	40 to <60 years old (*n* = 224) [a]	60 to 80 years old (*n* = 199) [a]
T (total)	PFOA	−11.8 (−22.2, −0.0) *	3.8 (−3.9, 12.0)	−5.0 (−12.7, 3.4)	4.3 (−5.7, 15.4)
	PFOS	−7.2 (−14.6, 0.9) *	1.9 (−4.5, 8.6)	−2.9 (−8.9, 3.5)	6.9 (−0.8, 15.1) *
	PFHxS	−6.5 (−12.2, −0.4) **	−2.8 (−8.2, 2.9)	−1.9 (−8.0, 4.6)	1.2 (−6.9, 9.9)
	PFNA	−10.5 (−19.2, −0.8) **	5.6 (−2.3, 14.1)	−6.3 (−14.1, 2.2)	5.4 (−3.3, 14.8)
T3 (free)	PFOA	1.5 (−2.1, 5.2)	0.2 (−1.3, 1.7)	−0.1 (−1.6, 1.3)	1.6 (0.0, 3.1) *
	PFOS	−1.5 (−3.8, 0.9)	0.2 (−1.1, 1.4)	−0.5 (−1.6, 0.6)	0.7 (−0.4, 1.9)
	PFHxS	−0.6 (−2.4, 1.2)	0.5 (−0.6, 1.6)	−0.1 (−1.2, 1.0)	0.8 (−0.4, 2.1)
	PFNA	−1.4 (−4.3, 1.5)	0.6 (−0.9, 2.1)	0.6 (−0.9, 2.1)	0.9 (−0.4, 2.2)
T3 (total)	PFOA	0.2 (−4.3, 5.0)	−0.8 (−3.4, 1.8)	−1.3 (−4.0, 1.4)	3.2 (0.6, 6.0) **
	PFOS	−2.8 (−5.7, 0.2) *	−0.6 (−2.7, 1.7)	−1.6 (−3.6, 0.4)	0.6 (−1.3, 2.6)
	PFHxS	−0.9 (−3.2, 1.4)	0.8 (−1.2, 2.7)	0.2 (−1.8, 2.3)	1.9 (−0.3, 4.1) *
	PFNA	−2.2 (−5.9, 1.5)	−0.8 (−3.4, 1.9)	−1.9 (−4.6, 0.9)	1.9 (−0.4, 4.2)
T4 (free)	PFOA	1.4 (−2.7, 5.7)	1.9 (−0.1, 3.9) *	1.8 (−0.6, 4.2)	−2.4 (−4.9, 0.3) *
	PFOS	−0.3 (−3.0, 2.5)	2.0 (0.4, 3.7) **	1.4 (−0.3, 3.3)	−0.8 (−2.7, 1.2)
	PFHxS	−0.3 (−2.4, 1.8)	1.0 (−0.5, 2.5)	0.6 (−1.2, 2.4)	−2.0 (−4.1, 0.2) *
	PFNA	−2.2 (−5.5, 1.2)	2.5 (0.5, 4.5) **	3.0 (0.6, 5.5) **	−2.4 (−4.6, −0.1) **
T4 (total)	PFOA	3.0 (−1.3, 7.5)	−0.8 (−3.3, 1.8)	0.3 (−2.5, 3.2)	−1.1 (−3.7, 1.5)
	PFOS	−0.2 (−3.1, 2.7)	−0.7 (−2.9, 1.5)	−0.4 (−2.5, 1.8)	−0.0 (−1.9, 1.9)
	PFHxS	−0.4 (−2.6, 1.8)	−0.9 (−2.8, 1.0)	0.5 (−1.6, 2.7)	−0.8 (−2.9, 1.3)
	PFNA	−3.3 (−6.7, 0.1) *	0.3 (−2.3, 3.0)	0.5 (−2.4, 3.5)	−0.5 (−2.7, 1.8)
TSH	PFOA	−14.7 (−26.6, −0.9) **	0.6 (−7.6, 9.5)	4.7 (−4.8, 15.1)	5.6 (−5.6, 18.2)
	PFOS	−6.5 (−15.5, 3.4)	−1.3 (−8.1, 6.0)	0.9 (−6.0, 8.4)	−0.1 (−8.1, 8.6)
	PFHxS	−3.0 (−10.2, 4.7)	0.3 (−5.8, 6.8)	6.3 (−1.0, 14.1) *	−0.3 (−9.2, 9.3)
	PFNA	3.0 (−9.1, 16.8)	−3.4 (−11.4, 5.3)	0.1 (−9.2, 10.3)	1.9 (−7.5, 12.1)

* $0.05 \leq p < 0.10$. ** $p < 0.05$.

Table S3. Percent change (95% CI) in serum hormone concentration associated with a doubling (100% increase) in serum PFAS concentration among adult males from NHANES 2011−2012 (adjusted results).

Hormone	PFAS	20 to <50 years old (*n* = 373) [a]	50 to 80 years old (*n* = 326) [a]
T (total)	PFOA	−1.1 (−5.1, 3.0)	5.4 (−1.5, 12.8)
	PFOS	−2.2 (−5.5, 1.2)	3.6 (−1.6, 8.9)
	PFHxS	−2.3 (−5.3, 0.8)	1.7 (−3.5, 7.2)
	PFNA	0.7 (−3.3, 4.9)	3.6 (−3.4, 11.1)
T3 (free)	PFOA	−0.1 (−1.1, 0.9)	−0.0 (−1.2, 1.2)
	PFOS	0.2 (−0.6, 1.1)	−0.2 (−1.1, 0.7)
	PFHxS	−0.2 (−0.9, 0.6)	−0.6 (−1.5, 0.3)
	PFNA	0.6 (−0.4, 1.6)	0.1 (−1.1, 1.4)
T3 (total)	PFOA	−0.6 (−2.3, 1.1)	1.3 (−1.0, 3.7)
	PFOS	−0.0 (−1.4, 1.4)	−0.2 (−1.9, 1.6)
	PFHxS	0.4 (−0.9, 1.7)	−0.7 (−2.5, 1.1)
	PFNA	0.4 (−1.3, 2.1)	0.5 (−1.9, 2.9)
T4 (free)	PFOA	−0.4 (−2.0, 1.2)	−0.3 (−2.4, 1.8)
	PFOS	−0.3 (−1.7, 1.1)	0.6 (−1.0, 2.2)
	PFHxS	−0.6 (−1.9, 0.7)	0.5 (−1.1, 2.2)
	PFNA	0.4 (−1.2, 2.1)	−0.4 (−2.5, 1.8)
T4 (total)	PFOA	−1.0 (−3.0, 1.0)	−1.5 (−4.0, 0.9)
	PFOS	−0.9 (−2.6, 0.7)	−0.8 (−2.6, 1.1)
	PFHxS	−0.1 (−1.6, 1.5)	−1.5 (−3.4, 0.4)
	PFNA	−0.4 (−2.3, 1.7)	−1.4 (−3.9, 1.1)
TSH	PFOA	0.9 (−5.0, 7.1)	−0.0 (−8.2, 9.0)
	PFOS	−1.7 (−6.6, 3.4)	−1.2 (−7.4, 5.3)
	PFHxS	−0.0 (−4.6, 4.7)	−1.0 (−7.4, 5.9)
	PFNA	0.4 (−5.4, 6.6)	1.5 (−7.1, 10.9)

[a] Adjusted for age (continuous), BMI (continuous), PIR (continuous), serum cotinine (continuous), and race/ethnicity (categorical).

Table S4. Percent change (95% CI) in serum hormone concentration associated with a doubling (100% increase) in serum PFAS concentration among adult females from NHANES 2011–2012 (adjusted results).

Hormone	PFAS	20 to <50 years old (n = 372) [a]	50 to 80 years old (n = 308) [a]
T (total)	PFOA	1.0 (−5.2, 7.6)	−1.9 (−9.7, 6.6)
	PFOS	0.8 (−4.2, 6.0)	5.2 (−1.2, 12.0)
	PFHxS	−2.8 (−7.3, 2.0)	−2.8 (−8.9, 3.8)
	PFNA	3.5 (−2.8, 10.4)	1.8 (−5.6, 9.8)
T3 (free)	PFOA	0.3 (−0.9, 1.5)	1.4 (0.1, 2.7) **
	PFOS	0.1 (−0.8, 1.1)	0.7 (−0.3, 1.7)
	PFHxS	0.4 (−0.5, 1.3)	0.6 (−0.4, 1.7)
	PFNA	0.7 (−0.5, 1.9)	1.1 (−0.1, 2.4) *
T3 (total)	PFOA	−0.5 (−2.5, 1.7)	2.6 (0.2, 5.0) **
	PFOS	−0.1 (−1.8, 1.6)	0.3 (−1.5, 2.1)
	PFHxS	0.7 (−0.9, 2.3)	2.0 (0.2, 3.9) **
	PFNA	−0.4 (−2.5, 1.7)	1.1 (−1.1, 3.3)
T4 (free)	PFOA	1.9 (0.2, 3.7) **	−1.3 (−3.4, 0.9)
	PFOS	2.0 (0.6, 3.4) **	−0.5 (−2.1, 1.1)
	PFHxS	1.1 (−0.2, 2.5)	−1.2 (−2.9, 0.5)
	PFNA	2.5 (0.8, 4.3) **	−1.1 (−3.0, 0.9)
T4 (total)	PFOA	0.7 (−1.4, 2.9)	−1.2 (−3.5, 1.3)
	PFOS	−0.1 (−1.8, 1.6)	−0.7 (−2.5, 1.1)
	PFHxS	−0.0 (−1.6, 1.6)	−0.5 (−2.3, 1.4)
	PFNA	0.8 (−1.3, 3.0)	−0.8 (−2.9, 1.4)
TSH	PFOA	1.0 (−5.5, 8.1)	3.2 (−6.3, 13.6)
	PFOS	−1.1 (−6.2, 4.4)	−0.3 (−7.4, 7.2)
	PFHxS	0.5 (−4.4, 5.8)	1.2 (−6.2, 9.1)
	PFNA	−3.7 (−9.9, 3.1)	4.2 (−4.5, 13.7)

[a] Adjusted for age (continuous), BMI (continuous), PIR (continuous), serum cotinine (continuous), and race/ethnicity (categorical). * $0.05 \leq p < 0.10$. ** $p < 0.05$.

© 2015 by the authors; licensee MDPI, Basel, Switzerland. This article is an open access article distributed under the terms and conditions of the Creative Commons Attribution license (http://creativecommons.org/licenses/by/4.0/).
